# UPF1 regulates myeloid cell functions and S100A9 expression by the hnRNP E2/miRNA-328 balance

**DOI:** 10.1038/srep31995

**Published:** 2016-08-30

**Authors:** Meike J. Saul, Stefan Stein, Manuel Grez, Per-Johan Jakobsson, Dieter Steinhilber, Beatrix Suess

**Affiliations:** 1 Department of Biology, Technical University Darmstadt, Schnittspahnstr. 10, 64287 Darmstadt, Germany; 2 Institute of Pharmaceutical Chemistry/ZAFES, Goethe University Frankfurt, Max-von-Laue-Str. 9, 60438 Frankfurt/M., Germany; 3 Georg-Speyer-Haus, Paul-Ehrlich-Str. 42-44, 60596 Frankfurt/M., Germany; 4 Department of Medicine, Rheumatology unit, Karolinska Institute, 17176 Stockholm, Sweden

## Abstract

UPF1 is a key player in nonsense mediated mRNA decay (NMD) but also involved in posttranscriptional gene regulation. In this study we found that UPF1 regulates the expression of genes with functions in inflammation and myeloid cell differentiation via hnRNP E2. The majority of the UPF1-regulated genes identified in monocytic cells contain a binding site for hnRNP E2 within 5′ UTR located introns with hnRNP E2 acting here as splicing regulator. We found that miRNA-328 which is significantly induced during monocytic cell differentiation acts independently from its gene silencing function as RNA decoy for hnRNP E2. One representative gene controlled by the hnRNP E2/miRNA-328 balance is S100A9 which plays an important role in cell differentiation and oxidative stress response of monocytes. Induction of miRNA-328 expression during cell differentiation antagonizes the blockade by hnRNP E2 which results in the upregulation of CD11b expression and ROS production in monocytic cells. Taken together, our data indicate that upregulation of miR-328 is responsible for the induction of hnRNP E2 target genes during myeloid cell differentiation.

Monocytes and macrophages play a central role in the innate immune system, responsible for the recognition and clearance of pathogens and dead cells. They are essential for the initiation and resolution of inflammation by phagocytosis, release of pro- and antiinflammatory cytokines, reactive oxygen species (ROS) and by regulation of the acquired immune system^1^,^2^. In response to specific stimuli, monocytes start to differentiate into macrophages. Subsequently, certain surface markers like CD14 are induced determining the differentiation state of monocytes^1^. Regulation of myeloid cell differentiation on the level of transcription has been studied extensively, however the impact of post-transcriptional regulation on this process is still less known.

The Up Frame Shift Protein 1 (UPF1) has originally been discovered as central component of the NMD pathway. However, in the last years it became obvious that UPF1 is not only important for the elimination of aberrant mRNAs harboring premature termination codons but is also involved in the regulation of gene expression controlling mRNA processing steps such as splicing, mRNA transport, translation or mRNA turnover^3^,^4^,^5^,^6^,^7^. A recent mass spectrometry-based proteomics study performed in our lab revealed that knockdown of UPF1 leads to multiple changes of the proteome in undifferentiated Mono Mac 6 (MM6) cells. Interestingly, the majority of the proteins downregulated by UPF1 knockdown returned to control levels during cell differentiation by TGFβ and calcitriol^8^. Pathway analysis demonstrated that c-Myc and granzyme A/B-mediated signaling pathways are highly associated with UPF1. Both pathways are correlated with myeloid cell differentiation and inflammatory responses^9^,^10^ which suggests an important regulatory function of UPF1 during myeloid cell maturation.

A detailed analysis of the genes downregulated by UPF1 knockdown led to the identification of a binding site for heterogeneous nuclear ribonucleoprotein (hnRNP) E2 in their 5′ UTR. HnRNPs are multifunctional RNA binding proteins involved in the processing pre-mRNA into mature mRNA, but are also important determinants of mRNA export, localization, transport and stability^11^. HnRNP E2, also known as αCP2 or polyC binding protein 2 (PCBP2) belongs to the class of minor hnRNP proteins^12^. While it is widely believed that hnRNPs (such as hnRNPE2) are involved in splicing^13^,^14^ some of them also mediate translational repression^15^. HnRNPs are ubiquitously expressed in all tissue types to varying levels. HnRNPs are predominantly nuclear at steady state; however, some of them are able to rapidly shuttle between the nucleus and the cytoplasm. Along with this, the multiple functionalities of hnRNP E2 as splicing regulator and translational repressor can be explained.

*C/EBP*α mRNA, a master regulator of myeloid cell differentiation, is a prominent example of translational inhibition by hnRNP E2. hnRNP E2 inhibits differentiation via binding to a C-rich motif in the 5′UTR of *C/EBP*α. Interestingly, the microRNA miR-328 can interact with hnRNP E2 independent from any known factors of the miRNA gene silencing machinery simply by acting as decoy of hnRNP E2 thus relieving translational inhibition of *C/EBP*α myeloid differentiation^16^.

Here we present data which indicate that the balance between hnRNP E2 and miR-328 controls the expression of many more genes involved in myeloid cell differentiation. We could show that UPF1 downregulates hnRNP E2 expression providing a link between UPF1 and hnRNP E2. Furthermore, we found that miR-328 is induced during myeloid cell differentiation and consequently, the balance between hnRNP E2 and miR-328 is altered leading to inhibition of hnRNP E2 and the concomitant upregulation of the expression of genes linked to myeloid cell maturation and function. A representative example of these proteins is the calcium binding protein S100A9 which was analyzed in detail. Taken together, our data demonstrate that cell functions such as ROS production and CD11b-mediated adhesion and migration is regulated by the hnRNP E2/miR-328 balance.

## Results

### mRNA expression analysis of downregulated proteins by UPF1 knockdown

**Figure 1:**
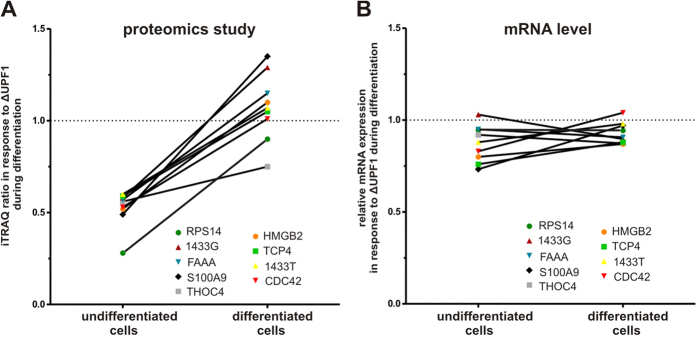
(**A**) Effect of UPF1 knockdown on UPF1 target protein expression in undifferentiated and differentiated MM6 cells. Cell differentiation was performed with TGFβ and calcitriol^8^. (**B**) Effect of UPF1 knockdown on mRNA expression of the indicated UPF1 target genes in 1 day differentiated and undifferentiated MM6 cells. The relative changes to control (set as 1) are given as the mean of three independent experiments.

In a mass spectrometry based proteomics study we identified a set of genes which are controlled by UPF1^8^. A subset of these proteins was downregulated in response to UPF1 knockdown in the microsomal fraction of undifferentiated cells and readjusted to control levels during differentiation (Fig. 1A and Table S1). Quantitative RT-PCR analysis of these genes revealed no significant changes in their mRNA expression (Fig. 1B), indicating that UPF1 influences gene expression on the posttranscriptional level.

### Identification of a C-rich motif within the 5′UTR of downregulated proteins by UPF1 knockdown

**Figure 2:**
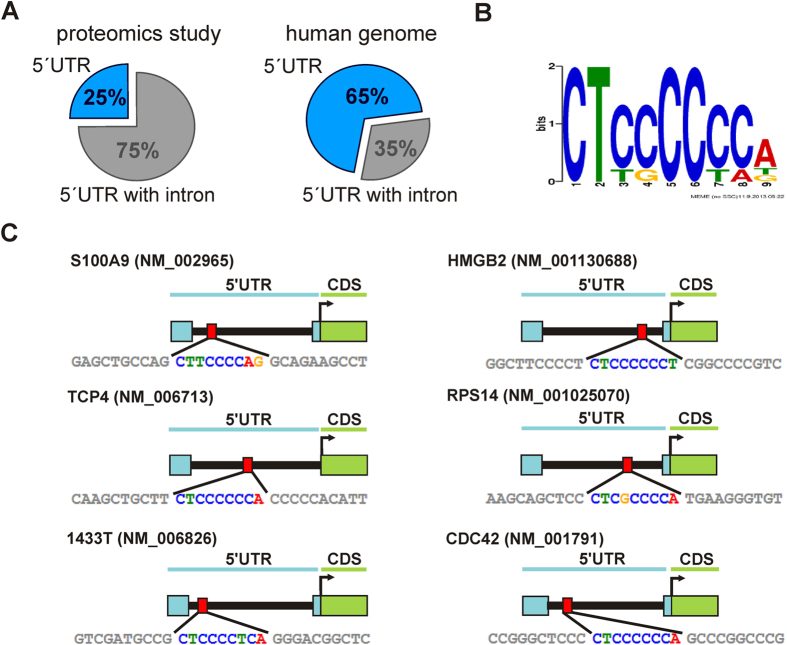
(**A**) Occurrence of 5′UTR introns in genes detected in the proteomics study compared to the composition of the entire human genome. (**B**) Conserved hnRNP E2 binding motif within the 5′UTR intron of genes coding for downregulated proteins by UPF1 knockdown (www.meme.sdsc.edu). The overall height of each stack indicates the sequence conservation at the respective position (measured in bits). (**C**) Localization of the C-rich sequence within the 5′UTR introns. The C-rich sequence is shown as red box, the intron as black line (not drawn to scale).

Close inspection of the gene structure of the candidates which are downregulated by UPF1 knockdown revealed that 75% of the genes contain an intron within their 5′UTR. This percentage is significantly higher than the average of all human genes with a 35% likelihood of introns at this position (Fig. 2A)^17^. This unusual high occurrence of 5′UTR introns suggested a search for potential regulatory sequences in this set of introns. We used the motif-based sequence analysis tool “MEME”^18^ and identified a C-rich consensus motif (CUCCCCC, Fig. 2B,C). In some introns the motif is present twice albeit its location is not conserved. This C-rich motif has already been described as a binding site for hnRNP E2 within the 5′UTR of *C/EBPα*^15^.

### UPF1 knockdown upregulates hnRNP E2 expression in undifferentiated and differentiated MM6 cells

**Figure 3:**
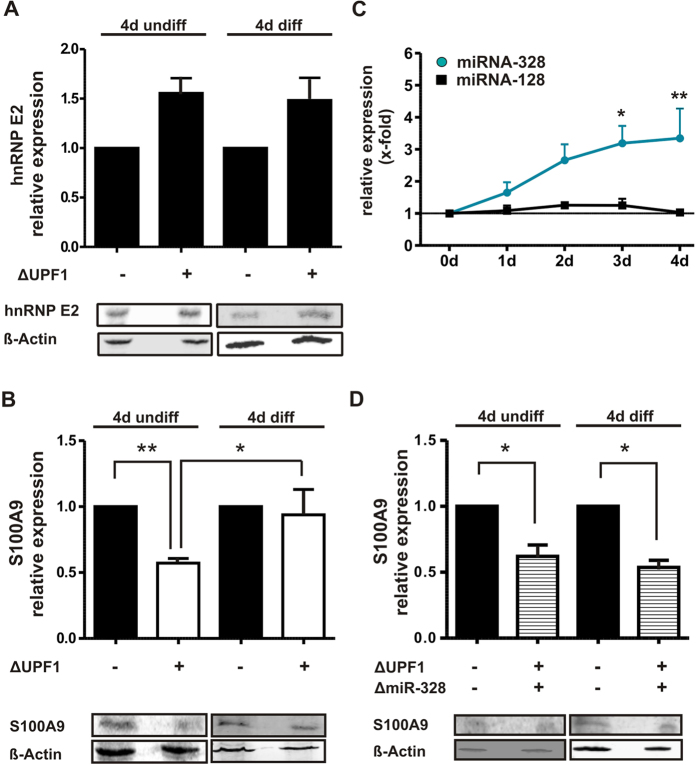
(**A**) Western blot analysis of hnRNP E2 protein expression in the microsomal fraction of MM6 and ∆UPF1 MM6 cells incubated with and without TGFβ (1 ng/ml) and calcitriol (50 nM) for 4 days. β-Actin served as loading control. (**B**) Western blot analysis of S100A9 protein expression of MM6 and ∆UPF1 MM6 cells and (**D**) MM6 cells and ∆miR-328 ∆UPF1 MM6 cells. The cells were incubated with and without TGFβ (1 ng/ml) and calcitriol (50 nM) for 4 days. β-Actin was used as loading control. The relative changes to control samples (set as 1) are given as mean + SEM of minimum three independent experiments; t-test, p* < 0.05; **p < 0.01. Blots are shown from one representative experiment. (**C**) Regulation of miRNA-328 and miRNA-128 expression during MM6 cell differentiation by calcitriol (50 nM) and TGFβ (1 ng/ml). After the indicated time points, RNA was extracted and miRNA expression was determined by qRT-PCR. The relative changes to day 0 are given as the mean + SEM of three independent experiments; two way ANOVA, Bonferroni post test, *p < 0.05, **p < 0.01.

The presence of the C-rich elements prompted us to investigate whether UPF1 mediates some of its cell type-dependent effects via hnRNP E2. We analysed the effect of UPF1 knockdown on hnRNP E2 expression in undifferentiated and differentiated MM6 cells by Western blot. Knockdown of UPF1 increases hnRNP E2 protein expression in undifferentiated and in 4 days differentiated MM6 cells (Fig. 3A). The data suggest that hnRNP E2 expression is downregulated by UPF1 in undifferentiated as well as in differentiated MM6 cells.

### Effect of UPF1 knockdown on S100A9 protein expression

A high percentage of the identified UPF1-regulated genes such as S100A9, CDC42, HMGB2 and members of 14-3-3 protein family are related to cell differentiation and inflammation^19^,^20^,^21^,^22^,^23^,^24^,^25^. In order to investigate the mechanism behind the UPF1 effects we selected S100A9, an important regulator for myeloid cell functions, for further analysis^26^. It is strongly upregulated during cell differentiation and regulates the inflammatory and migratory potential of myeloid cells. It functions as a damage-associated molecular pattern molecule which is actively secreted or released from necrotic cells in response to tissue injury or stress. Therefore, S100A9 is a significant functional representative for the identified differentiation related genes.

We validated the results of proteomics analysis where we saw a downregulation of S100A9 expression in undifferentiated but not in differentiated cells after UPF1 knockdown by Western blot. UPF1 knockdown caused a significant 50% reduction of S100A9 expression in undifferentiated but not in differentiated MM6 cells (Fig. 3B). These data confirm the results of the proteomics study showing that the effects of the UPF1 knockdown on S100A9 expression are dependent on the differentiation status of the MM6 cells.

### Reporter gene analysis of the S100A9 5′UTR

The next step was to determine whether S100A9 is regulated by hnRNP E2 via the C-rich element in the 5′-UTR intron. Therefore, we performed reporter gene assays with a reporter plasmid in which the 5′-UTR of S100A9 was cloned in front of a luciferase gene. In addition, we constructed a control plasmid in which the intron was deleted (S100A9Δint) and a plasmid with a mutated C-rich element (S100A9mut). Luciferase activity was analyzed in HeLa cells without and with UPF1 and hnRNP E2 knockdown, respectively. The siRNA-mediated knockdown of UPF1 or hnRNP E2 was verified by qRT-PCR and Western blot analysis. On mRNA level, the UPF1 and hnRNP E2 expression was reduced by 80% after 24h (Fig. S1A/B). On protein level, we observed a 50% to 70% reduction for UPF1 and hnRNP E2 expression (Fig. S1C/D).

**Figure 4:**
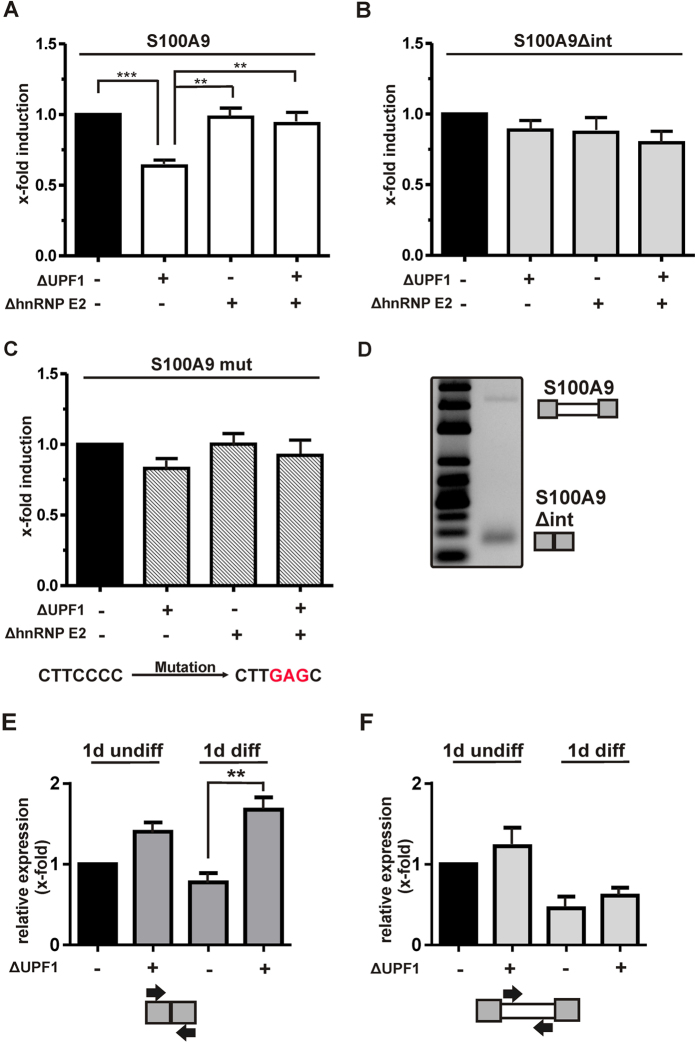
Luciferase reporter gene assays with S100A9 (**A**), S100A9Δint (**B**) and S100A9 mut (**C**) reporter plasmids. HeLa cells with knockdown of UPF1 and hnRNP E2 alone or in combination as indicated were transiently transfected. After 24 h, reporter gene activity was determined and normalized for transfection efficiency using the Dual-Glo^TM^ luciferase assay system. The relative changes in reporter gene activity are given as the mean + SEM of minimum three independent experiments; t-test, **p < 0.01, ***p < 0.001. (**D**) RT-PCR analysis of S100A9 5′UTR splicing in undifferentiated MM6 cells. PCR products obtained with specific primers flanking the S100A9 5′UTR intron were separated on a 3% agarose gel. (**E**) Effect of UPF1 knockdown on splicing of the S100A9 5′UTR intron in MM6 cells. ∆UPF1 or control MM6 cells were grown in standard medium or differentiated with TGFβ (1 ng/ml) and calcitriol (50 nM) for 1 day. Then, cells were harvested and total RNA was extracted and analyzed by qRT-PCR with primers spanning the 5′UTR intron (S100A9-5′UTR-F/R) and (**F**) with primers detecting specifically 5′UTR intron (S100A9-intron1-F/R). β-actin served as control. The relative changes to undifferentiated MM6 cells are given as the mean + SEM of at least three independent experiments; t-test, **p < 0.01.

The reporter gene assays revealed that the presence of the intron in the 5′UTR of S100A9 significantly increases luciferase reporter gene activity (Fig. S2). Luciferase activity of the S100A9 5′UTR construct was significantly reduced by UPF1 knockdown whereas hnRNP E2 knockdown alone had no influence on luciferase activity but abolished the UPF1 knockdown effect in the double knockdown experiment (Fig. 4A). Thus, hnRNP E2 inhibits reporter gene activity when UPF1 expression is low and the concomitant downregulation of hnRNPE2 restores reporter gene activity suggesting that hnRNP E2 functions as an inhibitor. In contrast to the S100A9 5′UTR construct, no influence of UPF1 or hnRNP E2 knockdown was observed when the intron was absent (S100A9Δint, Fig. 4B). Then, we mutated the C-rich motif of the S100A9 5′UTR construct (CTTCCCC to CTTGAGC). Similar to the S100A9Δint construct, the S100A9 mutant was unaffected by UPF1 or hnRNP E2 knockdown (Fig. 4C). These data suggest that UPF1 regulates S100A9 expression via the C-rich sequence and hnRNP E2. UPF1 downregulates hnRNP E2 expression (Fig. 3A) and reduces its inhibition of S100A9 expression via the C-rich sequence in the 5′UTR intron.

The mutation of the C-rich sequence within the intron suggests that hnRNP E2 may act as splicing regulator as previously shown for CD45^13^. An RT-PCR with specific primers targeting the 5′UTR region of S100A9 clearly shows the two splicing isoforms (Fig. 4D). We then performed qRT-PCR to quantify the splicing pattern of S100A9 5′UTR in MM6 cells with UPF1 knockdown. As shown in Fig. 4E, knockdown of UPF1 increases differentiation-dependently the 5′UTR splicing. The expression of the 5′UTR intron was not affected by UPF1 knockdown in undifferentiated and differentiated MM6 cells, respectively (Fig. 4F). These data indicate that hnRNP E2 act as intronic splicing silencer of S100A9 in a differentiation-dependent manner.

### MiRNA-328 inhibits hnRNP E2-mediated suppression of S100A9 expression in differentiated MM6 cells

The upregulation of hnRNP E2 in response to UPF1 knockdown is in line with the downregulation of S100A9 expression in undifferentiated MM6 cells. However, it cannot explain the observed upregulation of S100A9 expression and 5′UTR splicing in UPF1 knockdown cells during cell differentiation (Figs 1A, 3C and 4E) since there is no difference in hnRNP E2 protein expression in undifferentiated and differentiated MM6 cells (Fig. 3A). Recently, it was reported that miRNA-328 interacts with hnRNP E2 through a C-rich sequence thereby acting as a decoy^16^. Thus, we hypothesized that miRNA-328 might antagonize the hnRNP E2/S100A9 interaction by competing for binding to hnRNP E2.

First, we analyzed miRNA-328 expression in MM6 cells during differentiation with TGFβ and calcitriol for 4 days by qRT-PCR (Fig. 3C). Cell differentiation leads to a strong upregulation of miRNA-328 expression in a time-dependent manner. As control we used a randomly chosen miRNA, miRNA-128, whose expression was not affected by TGFβ and calcitriol. No significant changes in miRNA-328 expression were observed in MM6 cells upon UPF1 knockdown (Fig. S3) which demonstrates that miRNA-328 expression is not regulated by UPF1.

The strong differentiation-dependent upregulation of the miRNA328 suggests that it might inhibit repression by hnRNP E2 of the UPF1 regulated proteins through acting as a decoy as reported for C/EBPα^16^.

We knocked down miRNA-328 to analyze inhibition of hnRNP E2 activity by miRNA-328. A specific siRNA against the stem loop region of pre-miRNA-328 was used resulting in a significant reduction of miRNA-328 expression to nearly 40% in differentiated MM6 cells (Fig. S4). In order to investigate whether miRNA-328 is responsible for the prevention of S100A9 downregulation by UPF1 knockdown in differentiated cells, the effect of miRNA-328 knockdown on the expression of the hnRNP E2 target gene S100A9 was then analyzed in undifferentiated as well as differentiated ∆UPF1 cells. Knockdown of miRNA-328 leads to a significant reduction of S100A9 expression in undifferentiated as well as in differentiated ∆UPF1 MM6 cells (Fig. 3D).

The data suggest that upregulation of miR-328 expression during cell differentiation acts as hnRNP E2 decoy to prevent inhibition of hnRNP E2 target genes in differentiated cells. This is supported by the fact that upregulation of hnRNP E2 expression by UPF1 knockdown does not inhibit S100A9 expression in differentiated cells in the presence of miRNA-328 but when miRNA-328 is knocked down. Finally, it can be concluded that induction of miRNA-328 expression during cell differentiation is responsible for the inhibition of hnRNP E2-mediated repression of certain genes in differentiated cells.

### Influence of miRNA-328 on reactive oxygen species production and on the differentiation pattern of MM6 cells

Our data presented here suggest that UPF1 regulates the expression of genes containing a C-rich sequence in their 5′UTR via the balance between hnRNP E2 and the miRNA-328. Since a disproportionate high number of genes associated with myeloid cell functions are under the control of UPF1 (Fig. 1) we hypothesized that the hnRNP E2/miRNA-328 balance could be involved in the regulation of genes required for myeloid cell functions. Therefore, we investigated whether miR-328 knockdown has any influence on leukocyte functions such as the production of reactive oxygen species (ROS) or cell differentiation.

**Figure 5:**
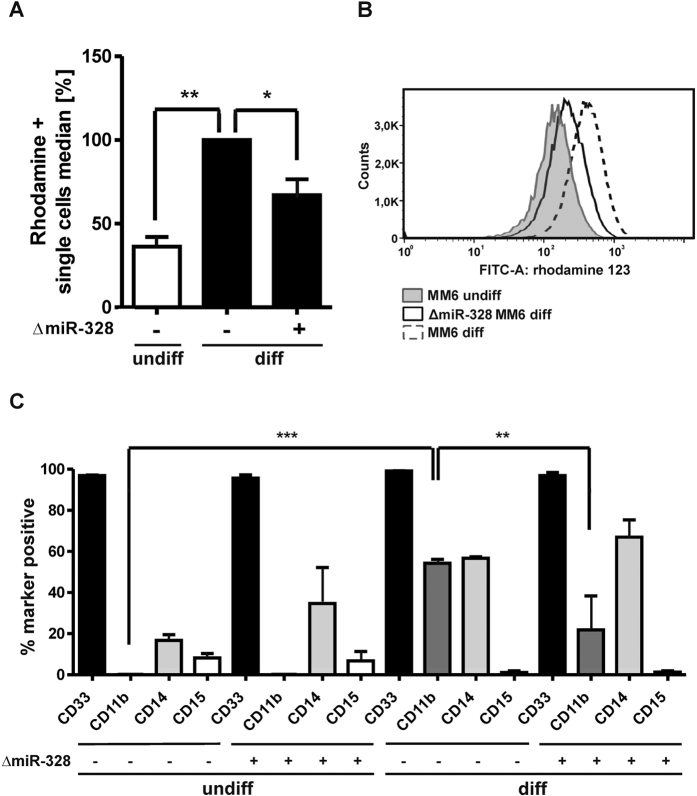
(**A**) Effect of cell differentiation and ∆miR-328 on the ROS production of MM6 cells. ROS production was determined with the dihydrorhodamine oxidation assay. The relative changes in ROS production to differentiated MM6 cells (set as 1) are given as mean + SEM of three independent experiments. (**B**) FACS histogram is shown from one representative experiment. (**C**) FACS analysis of CD33, CD11b, CD14 and CD15 expression in MM6 cells in response to cell differentiation and ∆miR-328. The relative changes are given as mean + SEM of three independent experiments, one way ANOVA test, Bonferroni post test, p* < 0.05, **p < 0.0, ***p < 0.001.

ROS production in undifferentiated and differentiated MM6 cells was analyzed using a dihydrorhodamine oxidation assay. The increase in intracellular ROS production during differentiation by TGFβ and calcitriol was significantly reduced by miRNA-328 knockdown (Fig. 5A,B).

The influence of miRNA-328 knockdown on cell differentiation was assessed by the determination of various monocytic surface markers, i.e. CD33, CD11b, CD14 and CD15 using FACS analysis (Fig. 5C). CD14 is a typical marker for mature peripheral blood monoytes. As expected, CD14 is upregulated in MM6 cells during differentiation^27^. It is slightly increased in undifferentiated and differentiated MM6 cells in response to miRNA-328 knockdown. CD33 is a monocytic surface marker, which can be found in immature myeloid cells^27^. No alteration of CD33 expression was observed in response to miRNA-328 knockdown. Furthermore, MM6 cells are also positive for CD15 expression, a monocyte counter-receptor for endothelial selectins^28^. The expression of CD15 was decreased during differentiation with TGFβ and calcitriol, but was not influenced by miRNA-328 knockdown. Finally, we measured CD11b expression which represents a marker for mature myeloid cells^29^. The analysis revealed a strong upregulation of the CD11b marker during MM6 cell differentiation which was significantly reduced by miRNA-328 knockdown (Fig. 5C). These data support a regulatory role of miRNA-328 during myeloid cell maturation.

## Discussion

Recently, the NMD factor UPF1 was identified as a critical regulator of gene expression in MM6 cells in a mass spectrometry based proteomics study^8^. Interestingly, expression of several proteins which were downregulated by UPF1 knockdown was readjusted to control levels during differentiation by TGFβ and calcitriol. Since there were no concomitant chances in the mRNA levels (Fig. 1B) it became obvious that UPF1 regulates the expression of these genes on posttranscriptional level, a function that might be independent from its role during NMD^8^,^30^ or it might be a consequence of secondary effects by NMD inhibition.

Many of the identified UPF1-regulated proteins possess an intron in the 5′-UTR containing a C-rich element as a common motif. The C-rich element is the binding motif for the RNA binding protein hnRNP E2. We found that hnRNP E2 expression is slightly decreased by UPF1 which provides the functional link between both proteins. Our reporter gene assay with the S100A9 5′UTR confirmed that the C-rich element in intron 1 mediates the UPF1 effects via hnRNP E2.

**Figure 6:**
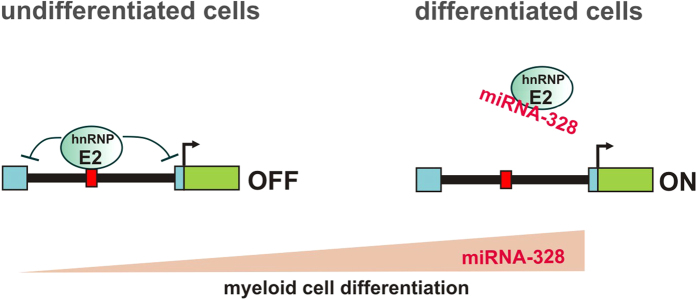
Regulation of gene expression during myeloid cell differentiation by the balance between hnRNP E2 and miRNA-328. Expression of hnRNP E2 target genes is induced by the upregulation of miR-328 expression during cell differentiation.

However, this mechanism could not explain the upregulation of S100A9 expression during cell differentiation since the hnRNP E2 levels in the controls as well as in the UPF1 knockdown cells which express elevated hnRNP E2 levels remain unchanged during differentiation, respectively. We found that miRNA-328 expression is strongly upregulated during MM6 cell differentiation (Fig. 3B) and by knockdown of miRNA-328 we could show that this miRNA acts as antagonist of hnRNP E2 thus preventing repression of its target genes in differentiated MM6 cells (Fig. 3D). The constant expression of hnRNP E2 and the cell differentiation-dependent increase in miRNA-328 expression suggest that the miRNA-328/hnRNP E2 balance is the critical determinant for hnRNP E2 activity and that upregulation of miRNA-328 expression during cell differentiation is responsible for the upregulation of hnRNP E2 target gene expression in differentiated cells (Fig. 6).

Moreover, we identified a disproportionate high ratio of potential hnRNP E2 target genes with functions related to inflammation and myeloid cell differentiation. For example the expression of Rho GTPase Cdc42 is associated with monocytic differentiation^31^ and it is essential for regulation between myelopoiesis and erythropoiesis^23^. Furthermore, two members of the 14-3-3 protein family 14-3-3G and 14-3-3T were identified as novel hnRNP E2/miR-328 targets. Members of this protein family inhibit TLR mediated cytokine induction^25^ and control monocytic migration and differentiation^32^,^33^. Additionally, we identified S100A9 as novel hnRNP E2 target gene, an important pro-inflammatory mediator in acute and chronic inflammation^34^. We chose the S100A9 for mechanistic investigations as it is prominently expressed in myeloid cells. Furthermore, S100A9 is an important protein for leukocyte functions which is mainly expressed in monocytes, early differentiated macrophages and neutrophils^26^. It forms a heterodimer with S100A8 and binds to RAGE and to TLR4, promoting inflammatory response in leukocytes^35^,^36^,^37^. Moreover, S100A9 interacts with the NADPH oxidase complex to increase ROS production in myeloid cells which in turn contributes to inflammation and differentiation of monocytes^38^,^39^.

We hypothesized that miRNA-328, by antagonizing hnRNP E2 function, controls myeloid cell differentiation and ROS production by upregulating of gene expression relevant for myeloid cell differentiation such as S100A9. Therefore, it was particularly interesting to evaluate the influence of miRNA-328 on the differentiation pattern and the cellular ROS production during myeloid cell maturation.

Indeed, we observed a significant reduction of CD11b expression in differentiated MM6 cells in response to miRNA-328 knockdown (Fig. 5C). CD11b plays an important role in the myeloid cell migration from the blood stream to the site of inflammation^40^,^41^,^42^ assuming that miRNA-328 directly modulates adhesive and migrating activities of monocytes. Furthermore, miRNA-328 knockdown causes a significant reduction of ROS production in differentiated MM6 cells (Fig. 5A,B), that might be at least in part related to the decreased S100A9 expression which is an important regulator of NADPH oxidase^43^. Furthermore, it is known that CD11b plays a critical role in the regulation of oxidative stress in monocytes^44^.

Taken together, our results demonstrate that the NMD factor UPF1 regulates genes with functions in myeloid cell differentiation via the balance between hnRNP E2 and miRNA-328. During monocyte maturation miRNA-328 is upregulated and antagonizes hnRNP E2 which then leads to increased ROS production as well as monocyte adhesion and migration.

## Materials and Methods

### Cell culture

MM6 cells were obtained from DSMZ (DSMZ no. ACC124) and grown in RPMI-1640 medium supplemented with 10% (v/v) fetal calf serum (FCS, Biochrom AG), 100 μg/ml streptomycin (PAA), 100 U/ml penicillin (PAA), 1× non essential amino acids (Sigma Aldrich), 10 μg/ml insulin, 1 mM oxaloacetate (AppliChem) and 1 mM sodium pyruvate (PAA). Cell culture was carried out in a humidified atmosphere of 5% CO_2_ at 37 °C. MM6 cells were differentiated with 1 ng/ml TGFβ and 50 nM calcitriol at 37 °C, 6% CO_2_.

The following stably transfected MM6 cells were used: MM6 control (MISSION shRNA plasmid CHCOO2, Sigma Aldrich) and MM6 ΔUPF1 (MISSION shRNA plasmid (NM_002911.2-2451s1c1, Sigma Aldrich)^45^.

HeLa cells were obtained from DSMZ (DSMZ no: ACC57). These cells were grown in Dulbecco’s modified Eagle’s medium (DMEM) supplemented with 10% (v/v) fetal calf serum, 100 μg/ml streptomycin, 100 U/ml penicillin. Cell culture was carried out in a humidified atmosphere of 5% CO_2_ at 37 °C.

### Plasmid constructs

S100A9 5′UTR reporter gene constructs were prepared using restriction enzymes and PCR methods. The complete 5′UTR of the S100A9 gene was PCR amplified using gDNA from MM6 cells as a template, the primers S100A9-Fwd (5′-AGTCGAGCTAG CAAACACTCTGTGTGGCTCCTCG-3′), S100A9-unspliced-Rev (5′- CTAGTACTCGAGC GTCTTGCACTCTGTCTGTGTAAT-3′) and *Phusion* polymerase (NEB). For amplification of spliced 5′UTR (S100A9Δint) the primers S100A9-Fwd and S100A9-spliced-Rev (5′-CTAGT ACTCGAGCGTCTTGCACTCTGTCAAAGC-3′) were used. The PCR fragments and the plasmid pGL4.10 (Promega) were digested by NheI and XhoI (NEB). The digested inserts were ligated in front of synthetic firefly luciferase (*luc2*) of pGL4.10 vector using T4 DNA ligase (NEB); the pGL4.10 plasmid was pretreated with antarctic phosphatase (NEB).

Mutation of the C-rich sequence was generated by site directed mutagenesis PCR of the construct S100A9 leading to the plasmid S100A9mut using the primers (GTAAGTGAGCTGCCAGCTTGAGCAGGCAGAAGCCTGCCTG) and (CAGGCAGGCTTC TGCCTGCTCAAGCTGGCAGCTCACTTAC), respectively, and *Pfu* polymerase (Fermentas). All plasmid sequences were confirmed by DNA sequencing.

### Transfection

24 h prior to transfection, HeLa cells were seeded at a density of 4 × 10^4 ^cells per well. 800 ng/well of S100A9-unspliced or S100A9-spliced luciferase reporter gene plasmid and 200 ng/well of pSV40-Rluc as internal standard were transfected using Lipofectamine2000^®^ (Invitrogen) according to manufacturer′s instructions. For co-transfection with siRNAs, 200 ng/well of reporter gene construct, 200 ng/well of pSV40-Rluc and 20 pmol/well siRNA were used for transfection with Lipofectamine2000. After 24 h, reporter gene activity was determined with the Dual-Glo™ Stop and Glow Luciferase Assay system following the manufacturer’s protocol (Promega) and measured with a Tecan infinite^®^ M200 reader. Renilla luciferase activity was used to normalize the luciferase activity to the transfection efficacy.

### RNA extraction and real-time quantitative RT-PCR

Total RNA was extracted with RNeasy Mini kit (Qiagen) according to manufacturer’s instructions. Residual DNA was removed by on-column DNAse digestion using RNase-Free DNase Set (Qiagen). 1 μg RNA was used for cDNA synthesis using High Capacity RNA-to-cDNA Kit (Applied Biosystems). Real-time quantitative PCR (qRT-PCR) was performed in Applied Biosystems StepOne Plus^TM^ Real-Time PCR System (Applied Biosystem) using Power SYBR Green PCR Master Mix (Applied Biosystems). Fold inductions were calculated using 2^(−ΔΔCt)^-values. Primer sequences are given in Table 1.

### RT-PCR

0.5 μl cDNA was used for RT-PCR using 0.02 U/μl Q5^®^ High-Fidelity DNA Polymerase (NEB) according to the manufacturer’s instructions with 4% DMSO addition. The primers S100A9 RT-PCR-F (CACTCTGTGTGGCTCCTCG) and S100A9 RT-PCR-R (CGTCTTGC-ACTCTGTCTG) were used for the amplification of S100A9 5′UTR.

### RNA interference

UPF1 and hnRNP E2 were transiently depleted using siRNA oligonucleotides. 24 h prior to transfection, HeLa cells were seeded at a density of 4 × 10^4 ^cells per well. 20 pmol/well siRNA oligonucleotides were transfected using Lipofectamin2000^®^ (Invitrogen) according to manufacturer′s instructions. For UPF1 knockdown, MISSION^®^ siRNA SASI_Hs01_00101018 (Sigma Aldrich) and for hnRNP E2 knockdown, MISSION^®^ siRNA SASI_Hs01_00319507 (Sigma Aldrich) were used. As control, a siRNA against GFP was designed (5′-UCUCUCACAACGGGCAUUU-3′). Cells were harvested 24 h after transfection. The efficiency of UPF1 or hnRNP E2 knockdown was assessed by qRT-PCR and Western blot.

### MicroRNA quantification using stem loop real-time RT-PCR

100 ng freshly isolated RNA were transcribed into cDNA using miRNA specific stem-loop primer. qRT-PCR using Universal Probe library probe #21 (Roche Diagnostics), TaqMan Universal PCR Master Mix 2 x (Applied Biosystem) and a miRNA specific primer was performed on in Applied Biosystems StepOne Plus^TM^ Real-Time PCR System (Applied Biosystem). Specific primers were designed for the specific amplification of miRNA-328, miRNA-128 and the loading control U48.

Stem loop primer miRNA-328: GTTGGCTCTGGTGCAGGGTCCGAGGTATTCGCAC CAGAGCCAACACGGAA, stem loop primer miRNA-128: GTTGGCTCTGGTGCAGGGTCCGAGGTATTCGCACCAGAGCCAACCTGTTC, stem loop primer snoRNA-U48: GTTGGC TCTGGTGCAGGGTCCGAGGTATTCGCACCAGAGCCAACGGTCAG, forward primer miRNA-328: GCTGGCCCTCTCTGCCC, forward primer miRNA-128: CCGGTCACAGTGAA CCGGT, forward primer snoRNA-U48: GAGTGATGATGACCCCAGGTAA, universal primer: GTGCAGGGTCCGAGGT. Stem loop reverse transcription and real time RT-PCR were preformed as described previously^46^. Fold inductions were calculated using 2^(−ΔΔCt)^-values.

### miRNA-328 knockdown in MM6 cells

Using siDesign Center (GE Dharmacon) a specific siRNA was designed against the loop region of pre-miRNA-328 to downregulate miRNA-328 expression. 2 pmol/μl of the 3′-cholesterol-tagged ON TARGET siRNA-pre-miR-328 (GGGAGAAAGUGCAUACAGC-3′-Chl) or control siRNA (5′-UCUCUCACAACGGGCAUUU-3′-Chl) was directly added to MM6 cell culture medium. Both siRNAs were synthesized by GE Dharmacon. The efficiency of miR-328 knockdown was assessed by stem loop real time RT-PCR in 4 days differentiated MM6 cells.

### Western blot analysis

The efficiency of UPF1 and hnRNP E2 knockdown in HeLa cells was assessed by Western blot analysis. Cells were lysed in T-PER™ tissue protein extraction reagent (Life technologies) for 30 min at 4 °C. The protein content was determined by Bradford assay (BioRad Laboratories). Western blot analysis of the cell lysates and microsomal fraction was performed as previously described^8^. The following antibodies were used to stain the blots: hnRNP E2 (ab77323, Abcam), UPF1 (antiserum was generously supplied by Jens Lykke-Andersen, University of California, San Diego), S100A9 (ab92507, Abcam) and β-actin (sc-1616, Santa Cruz Biotechnology).

### Dihydrorhodamine 123 assay

About of 5 × 10^5 ^MM6 cells were resuspended in 2.5 ml of Hanks Balanced Salt Solution with Ca^2+^/Mg^2+^ (HBSS, Invitrogen), 0.5% bovine serum albumin (PAA), 1000 U catalase (Sigma), 7.5 mM glucose (Roth) and incubated for 5 min at 37 °C. Then, dihydrorhodamine 123 (DHR, Sigma) was added to a final concentration of 0.29 μM, incubated for further 15 min at 37 °C, kept on ice for 30 min and finally analyzed using FACS Canto II (Becton Dickinson).

### FACS analysis of surface marker

About 1 × 10^5 ^MM6 cells were incubated for 30 min at room temperature with antibodies for CD11b (CD11b-PECy7, eBioscience), CD14 (CD14-APC, eBioscience), CD15 (CD15-eFluor 450, eBioscience), CD33 (CD33-PE P67.6, eBioscience), FVD eFluor^®^ (eBioscience) or with the proper control IgGs. The optimal concentration for each antibody was adjusted according to manufacturer’s instructions. To block unspecific binding of antibodies, human Fc Block (Miltenyi) was added to the cell suspension. After washing with PBS, FACS analysis was performed using FACS Canto II (Becton Dickinson).

### Statistics

Results are given as mean + SEM of at least three independent experiments. Statistical analysis was carried out by Student’s paired or unpaired t-test (two-tailed), one way ANOVA or respectively for the time course experiment two way ANOVA, Bonferroni post test was used. Differences were considered as significant for *p* < 0.05 (indicated as *p < 0.05, **p < 0.01 and ***p < 0.001) using GraphPad Prism 5.0.

## Additional Information

**How to cite this article**: Saul, M. J. *et al.* UPF1 regulates myeloid cell functions and S100A9 expression by the hnRNP E2/miRNA-328 balance. *Sci. Rep.* **6**, 31995; doi: 10.1038/srep31995 (2016).

## Supplementary Material

Supplementary Information

## Figures and Tables

**Table 1:** Primer sequences used for qRT-PCR.

Primer	Sequence
Actin-F	CGGGACCTGACTGACTACCTC
Actin-R	CTTCTCCTTAATGTCACGCACG
CDC42-F	TGCACTTACACAGAAAGGCC
CDC42-R	CTTCTTCGGTTCTGGAGGCT
1433T-F	CGGTGCTGGAATTGTTGGAT
1433T-R	TTCAGCAAGGTACCGGAAGT
HMGB2-F	CTAAAAGGCCACCATCTGCC
HMGB2-R	GATAGGCCAGGGTGTTCACT
TCP4-F	TCAAGCTCTTCTGGCAGTGA
TCP4-R	GCTCTCGAAGTCTCACCTGT
S100A9-5′UTR-F	CACTCTGTGTGGCTCCTCG
S100A9-5′UTR-R	GTTCCAGCTGCGACATTTTG
S100A9-intron1-F	AGCTGCCAGCTTCCCCAGG
S100A9-intron1-R	GCTGTCAAGCTTCTTTGACAC
S100A9-cds-F	GTGCGAAAAGATCTGCAAAATTT(*)
S100A9-cds-R	GGTCCTCCATGATGTGTTCTATGA (*)
THOC4-F	AGGCCTGCACAGAGCGTAA
THOC4-R	TCCAGCGCCACGGTTT
RPS14-F	TCGGGCGGATTGAGGAT
RPS14-R	TTCCTGCGAGTGCTGTCAGA
1433G-F	GGAGCGCTACGACGACATG
1433G-R	AGTGGCTCATTCAGCTCTGTCA
FAAA-F	GAGCCAGGCGGCTACCAT
FAAA-R	TGCAGCATCGTCCAGTACATG
hnRNP E2-F	GAACTCACCATTCCAAACGATTT
hnRNP E2-R	TTGATTTTGGCGCCTTGAC
UPF1-F	CCTTCCCATCCAACATCTTC
UPF1-R	AACATCGGTTTATCGGGTTG

(*) Primer sequence adopted from^47^.
