# Supplementary material for: UPF1 regulates myeloid cell functions and S100A9 expression by the hnRNP E2/miRNA-328 balance
Source: Sci Rep. 2016 Aug 30;6:31995. doi: 10.1038/srep31995 (PMC5004132; doi:10.1038/srep31995)
Supplement: Supplementary Information [file srep31995-s1.pdf]

Supplementary information for:

UPF1 regulates myeloid cell functions and S100A9 expression by the hnRNP E2/miRNA-328 balance

Meike J. Saul[1,2], Stefan Stein[3], Manuel Grez[3], Per-Johan Jakobsson[4], Dieter Steinhilber[2*], Beatrix Suess[1*]

[1] Department of Biology, Technical University Darmstadt, Schnittspahnstr. 10, 64287 Darmstadt, Germany

[2] Institute of Pharmaceutical Chemistry/ZAFES, Goethe University Frankfurt, Max-von-Laue-Str. 9, 60438 Frankfurt/M., Germany

[3] Georg-Speyer-Haus, Paul-Ehrlich-Str. 42-44, 60596 Frankfurt/M., Germany

[4] Department of Medicine, Rheumatology unit, Karolinska Institute, 17176 Stockholm, Sweden

*Corresponding authors: Beatrix Suess (bsuess@bio.tu-darmstadt.de), Dieter Steinhilber (steinhilber@em.uni-frankfurt.de)

Table S1 iTRAQ ratio of downregulated proteins in the microsomal and soluble fraction according to [8].

	Microsomal fraction		Soluble fraction	
**Description**	**MM6 ΔUPF1/ MM6**	**MM6 diff Δ UPF1/ MM6 diff**	**MM6 ΔUPF1/ MM6**	**MM6 diff Δ UPF1/ MM6 diff**
40S ribosomal protein S14 [RPS14]	0,28	0,90	0,88	1,04
THO complex subunit 4 [THOC4]	0,56	0,75	1,07	0,97
14-3-3 protein gamma [1433G]	0,58	1,29	1,01	0,92
Fumarylacetoacetase [FAAA]	0,57	1,15	0,91	0,93
S100A9 [S10A9]	0,49	1,35	1,08	1,34
High mobility group protein B2 [HMGB2]	0,52	1,10	1,09	0,93
14-3-3 protein theta [1433T]	0,60	1,07	0,85	1,12
Activated RNA polymerase II transcriptional coactivator p15 [TCP4]	0,59	1,05	0,95	0,99
Cell division control protein 42 homolog [CDC42]	0,53	1,01	0,96	1,01

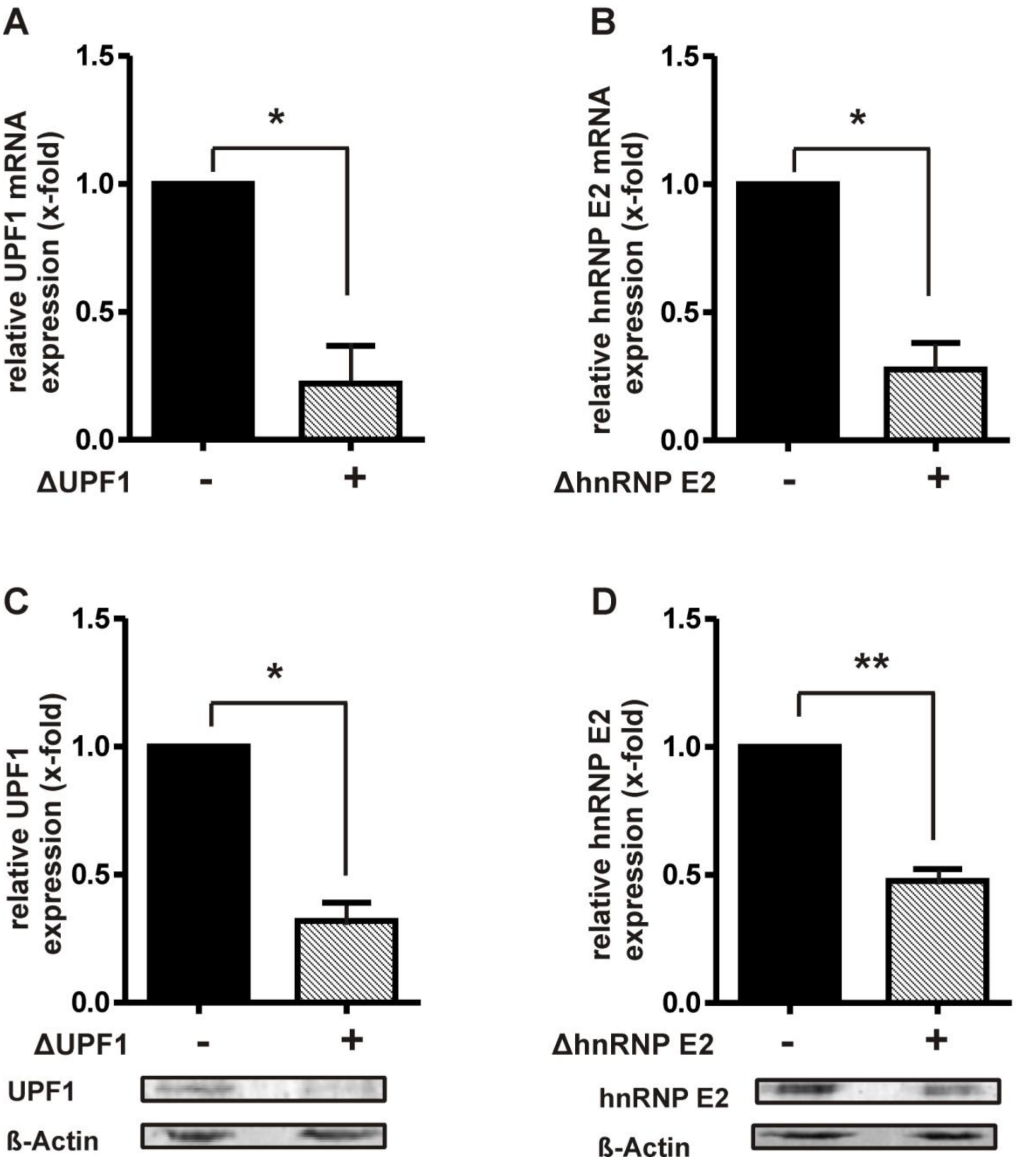

Figure S1 qRT-PCR analysis of UPF1 and hnRNP E2 mRNA expression in HeLa cells with and without knockdown of (A) UPF1 or (B) hnRNP E2 (24h). The relative changes to control (set as 1) are given as the mean + SEM of three independent experiments. Western blot analysis of UPF1 and hnRNP E2 expression in HeLa cells with and without knockdown of (C) UPF1 or (D) hnRNP E2 (24h). The relative changes to control (set as 1) are given as the mean + SEM of three independent experiments, *$p < 0.05$, **$p < 0.01$.

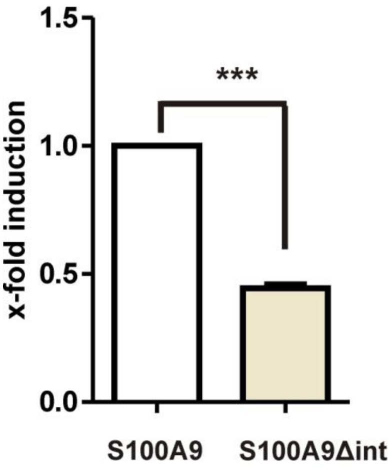

Figure S2 Luciferase reporter gene assay with S100A9 and S100A9Δint reporter plasmids. HeLa cells were transiently transfected with the indicated reporter plasmids. After 24 h, reporter gene activity was determined and normalized for transfection efficiency using the Dual-Glo™ luciferase assay system. The relative changes in reporter gene activity are given as the mean + SEM of minimum three independent experiments; t-test, ***$p < 0.001$.

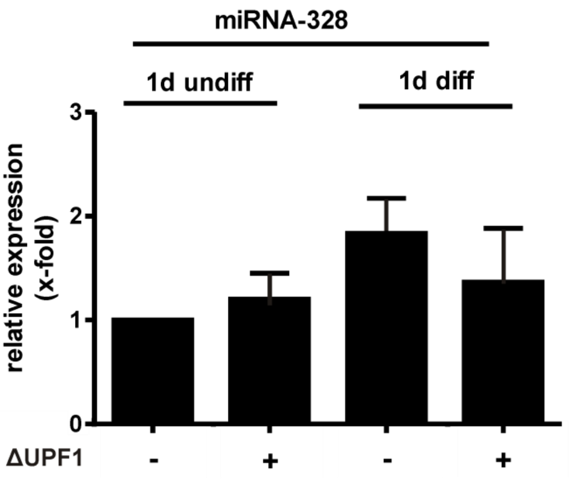

Figure S3 qRT-PCR analysis of miRNA-328 expression in 1 day differentiated and undifferentiated MM6 and ΔUPF1 MM6 cells. The relative changes to undifferentiated control are given as the mean + SEM of three independent experiments.

Figure S4

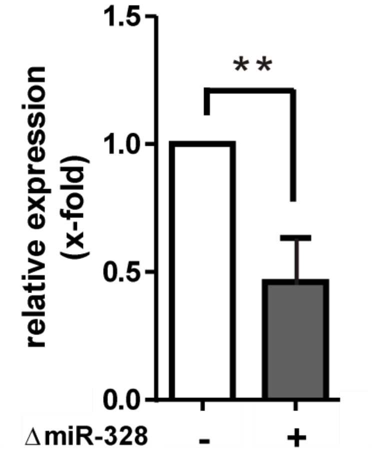

Figure S4 Quantification of miRNA-328 knockdown efficiency using qRT-PCR analysis. miRNA-328 knockdown was generated using a specific siRNA against pre-miR-328 in 4 days differentiated MM6 cells. The relative changes to control are given as the mean + SEM of four independent experiments; t-test **p < 0.01.
